# Retraction Note: Phytochemical studies of Veitchia arecina Becc. leaves with evaluation of in-vitro, in-vivo anti-inflammatory, and wound healing properties in rats

**DOI:** 10.1007/s10787-026-02261-y

**Published:** 2026-05-15

**Authors:** Radwa S. Emad, Rasha A. Attia, Mona Fekry, Nesreen M. I. M. Elkomy, Tarek M. Ibrahim, Amal A. Al-Gendy

**Affiliations:** 1 Faculty of Pharmacy, Department of Pharmacognosy, Zagazig University, P.O. Box 44519, Zagazig, Egypt; 2 Pharmacognosy Department, College of Pharmacy, Uruk University, Uruk, Iraq; 3 Faculty of Pharmacy, Pharmacology and Toxicology Department, Zagazig University, Zagazig, Egypt; 4 Faculty of Pharmacy, Pharmaceutics Department, Zagazig University, Zagazig, Egypt

**Retraction Note to: Inflammopharmacology (2025) 33:5525–5543** 10.1007/s10787-025-01802-1

The Editor-in-Chief has retracted this article. After concerns were raised about the animal experiments conducted in this research, an investigation by the Publisher found significant ethical concerns. Specifically, the welfare of the animals and methodologies used in this study do not appear to adhere to ethical guidelines. Upon request, the authors provided the ethics approval and the experimental protocol for this study. However, the documents did not provide a satisfactory response to the concerns raised while also appearing to indicate breaches of the Journal’s editorial policies for research involving animals.

Therefore, the Editor-in-Chief no longer has confidence in the results and conclusions of this article.

Author Radwa Sami, on behalf of all authors, disagrees with this retraction.

